# Pseudodominant inheritance in paraplegin (*SPG7*)-associated CPEO–ataxia syndrome

**DOI:** 10.1007/s10048-026-00955-7

**Published:** 2026-10-28

**Authors:** Weber Liu, Serge Geara, Simon Rowe, Carolyn Mary Sue

**Affiliations:** 1 Neuroscience Research Australia, Randwick, NSW 2031 Australia; 2 Faculty of Medicine and Health, University of New South Wales, Sydney, Australia; 3 Department of Neurology, Prince of Wales Hospital, Sydney, NSW Australia; 4 Faculty of Medicine and Health, University of Sydney, Sydney, NSW Australia

**Keywords:** Pseudodominant inheritance, Paraplegin, *SPG7*, Mitochondrial disease, Cascade testing

## Abstract

*SPG7* encodes paraplegin, a mitochondrial AAA-metalloprotease critical for inner mitochondrial membrane protein quality control. Pathogenic variants most commonly cause autosomal recessive hereditary spastic paraplegia (HSP) but its phenotypic spectrum includes cerebellar ataxia, progressive external ophthalmoplegia, myopathy and parkinsonism.

We report a 47 year old man presenting with a progressive external ophthalmoplegia, ptosis, progressive gait disturbance, and spasticity. Ten-year follow-up of this individual showed slowly progressive spastic ataxia with ocular motor involvement and neurogenic bladder. His mother and maternal aunt both had marked spasticity, ataxia, ptosis and external ophthalmoplegia. Genetic testing demonstrated compound heterozygous pathogenic variants in paraplegin in this individual. Genetic testing in his mother and aunt revealed homozygous canonical splice-site variants (c.2182–2 A > G). Cascade testing of his asymptomatic father showed a common pathogenic missense variant p.(Ala510Val) in the same gene.

This family demonstrates a pseudodominant inheritance pattern, instead of the expected autosomal recessive trait that applies to paraplegin. We highlight diagnostic pitfalls in relying on family history and assumed inheritance traits. We emphasise the importance of segregation analysis and comprehensive genomic testing. Recognition of pseudodominance inheritance has important implications for genetic counselling, family planning and cascade testing.

## Background

The *SPG7* gene encodes for paraplegin, a mitochondrial protein whose enzymatic activity is crucial to mitochondrial proteostasis and mitophagy [1]. Pathogenic loss-of-function variants of *SPG7* are typically inherited in an autosomal recessive pattern, and result in the accumulation of misfolded mitochondrial proteins and subsequent dysfunction. The phenotypic spectrum of *SPG7* disease resembles that of mitochondrial disease, with reports of spastic paraparesis, cerebellar ataxia and progressive external ophthalmoplegia [2, 3]. Given high background carrier frequency in certain European populations [4], *SPG7* disease may be vertically transmitted in a manner resembling an autosomal dominant pattern, termed ‘pseudodominant transmission’, when an asymptomatic parent unknowingly passes on a second pathogenic variant. Here we describe a mother and son with apparent vertical transmission of *SPG7* and subsequent identification of a paternal carrier through cascade testing.

## Case 1

**Fig. 1:**
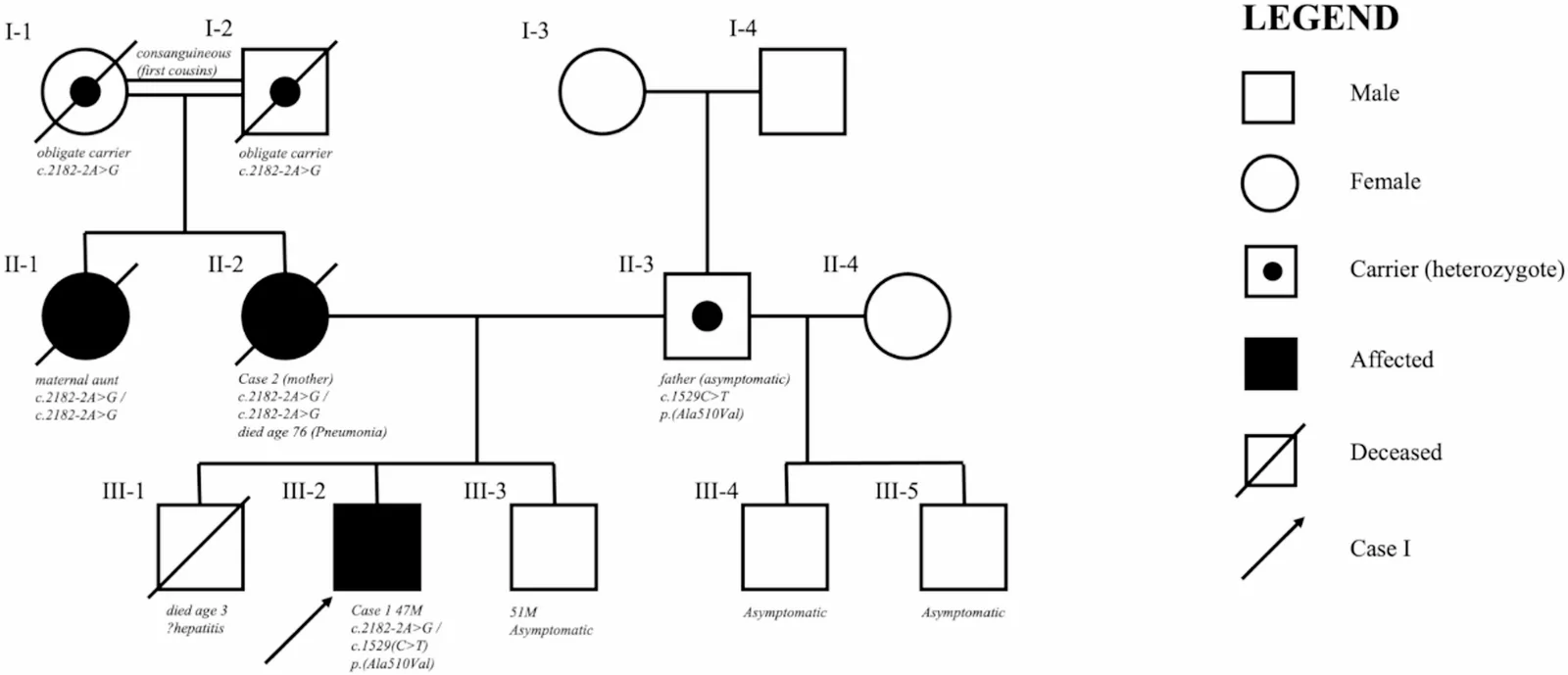
Family pedigree. Case 1(III-2) is denoted with an arrow, and Case 2(II-2) is their affected mother. Generations are labelled with Roman numerals (I–III) and individuals with Arabic numerals. Squares denote males and circles females; filled symbols indicate clinically affected individuals and a central dot an asymptomatic heterozygous (carrier) individual. A diagonal line denotes a deceased individual and the double horizontal line a possible consanguineous (first-cousin) union. Case 1(III-2) is compound heterozygous for *SPG7* c.2182–2A>G and c.1529C>T p.(Ala510Val); his mother (Case 2, II-2) and maternal aunt (II-1) are homozygous for *SPG7* c.2182–2A>G, and the maternal grandparents (I-1, I-2) are obligate carriers; Case 1’s asymptomatic father (II-3) is a heterozygous carrier of p.(Ala510Val) identified on cascade testing

A 47-year-old gentleman presented with a progressive lower limb ataxia and stiffness developing over an 18-month period. He recalls progressive loss of balance and incoordination while exercising, with medial scuffing of his shoes from age 40. He was born in New Zealand to non-consanguineous parents of Danish and Irish heritage. His mother (Case 2) and maternal aunt were previously known to our service and diagnosed with homozygous mutations in paraplegin (*SPG7*) with genetic confirmation prior to Case 1’s symptom onset. His father, two full siblings and two paternal half-brothers were asymptomatic (Fig. 1).

**Fig. 2:**
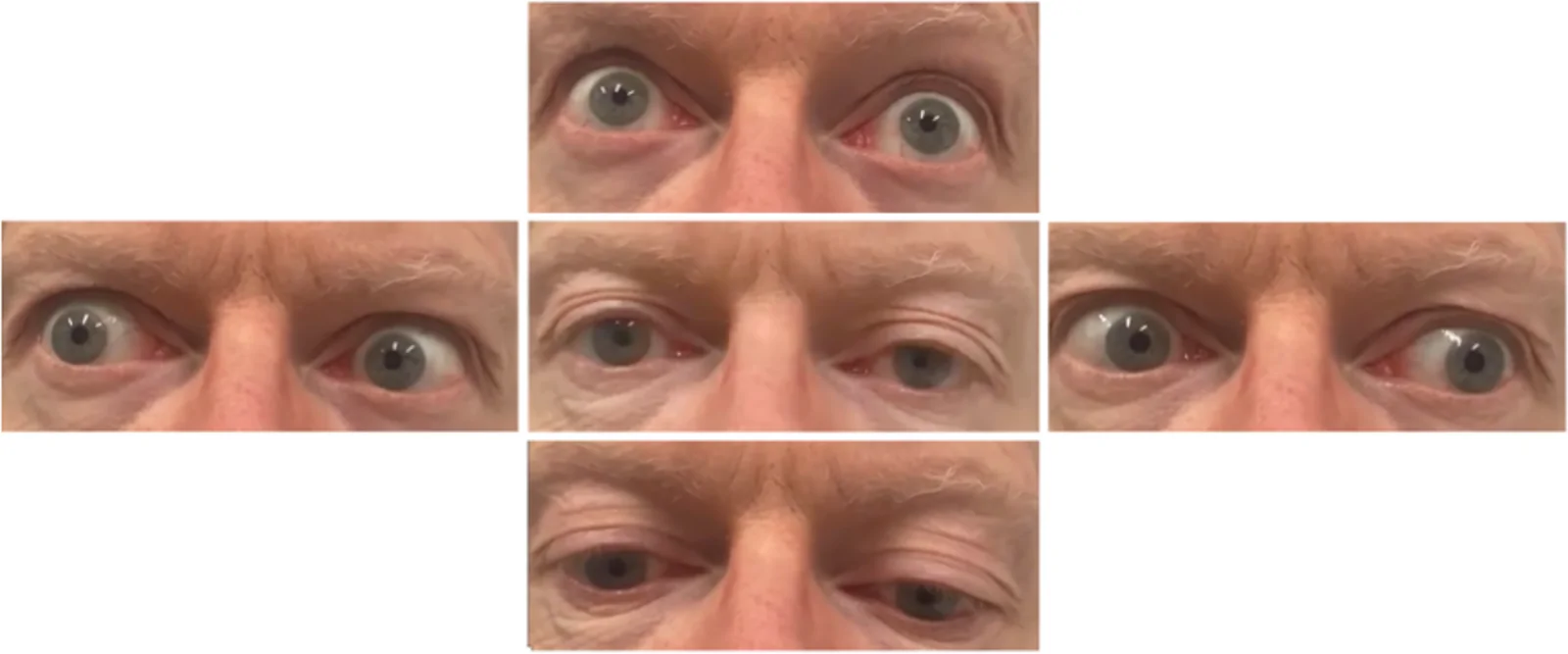
Eye movements of Case 1 at 10 years followup. Central panel indicates resting gaze, with horizontal and vertical eye movements shown. Note baseline left sided ptosis greater than right with horizontal and vertical gaze restrictions

On initial examination, the patient demonstrated normal cognition and non-dysarthric speech. He exhibited generalised progressive ophthalmoplegia (PEO) (Fig. 2) with asymmetric ptosis primarily affecting his left eye without visual axis impairment, and upper facial weakness. Saccadic slowing was noted, with normal visual acuity and fundoscopy. He had bilateral lower limb hypertonia and hyperreflexia without weakness or heel-shin ataxia. No sensory deficits were noted on examination. He mobilised with a scissoring gait. Tandem gait was difficult beyond three steps. Upper limb examination was otherwise normal.

Four years after initial presentation, he developed upper limb involvement with hypertonia and dysmetria, progressive horizontal saccadic slowing, and a neurogenic bladder. After 7 years, progressive decline in mobility had resulted in him giving up bushwalking, and new vertical gaze restriction was evident. At 10 years follow up, he can walk unaided but demonstrates a spastic ataxic gait with circumduction and complete impairment of tandem gait, without truncal ataxia. Proximal power remains unaffected. He has not demonstrated any bulbar signs.

Magnetic Resonance Imaging (MRI) of his brain and orbits was unremarkable and did not demonstrate cerebellar atrophy. Given his mother’s genotype was known and was diagnosed with an autosomal recessive transmitted disease, next-generation sequencing (PathWest Neuromuscular gene panel) was performed and demonstrated compound heterozygous pathogenic variants in *SPG7* c.2182–2A>G and c.1529C>T p.(Ala510Val). Cascade testing of his asymptomatic father revealed a heterozygous *SPG7* c.1529C>T p.(Ala510Val) variant.

## Case 2: Mother of Case 1

A 60 year-old-female previously known to our service initially presented with progressive ataxia and gait disturbance 20 years earlier to Case 1(Fig. 1, II-2). She reported a history of progressive gait disturbance from age 21, eventually requiring a single point stick for mobility by 35, and years of blepharospasm, progressive bilateral ptosis and cervical dystonia from her 40s. She denied seizures, hearing loss or other significant diagnoses. She was born to parents of Irish heritage with possible parental consanguinity. Her parents were reportedly asymptomatic, however her sister (Fig. 1, II-1) had a similar gait disturbance. Her sister at this time was 53 years and had required a single-point stick and wheelchair by 25 and 30 respectively. Her sister had also demonstrated bilateral ptosis, PEO, dysarthria and dysphagia. Case 2 had three children, including Case 1. One child reportedly died of fulminant hepatitis at age 3, and the other child is currently aged 51 and asymptomatic. She has since passed away at age 76 of pneumonia, and her sister has also passed away from colorectal cancer.

From her records of her last examination at age 75, she had cognitive impairment and dysarthric speech. She demonstrated bilateral ptosis, external ophthalmoplegia and optic disc atrophy without pigmentary retinopathy. She had truncal ataxia with a scissoring gait, global hyperreflexia with spasticity and finger-nose ataxia. Plantar responses were extensor. At this point she was wheelchair bound and required assistance for all transfers and activities of daily living.

MRI of the brain demonstrated minor cerebellar atrophy without a lactate peak on spectroscopy. A muscle biopsy of her right vastus lateralis at age 56 demonstrated abnormal fibre type grouping suggesting a neurogenic process, and electron microscopy noted abnormal mitochondria containing intracristal crystals and ring crystae, evidencing mitochondrial myopathy. Bloodwork was normal, without evidence of renal dysfunction, liver enzyme abnormalities, elevated HbA1c or lactate. Electrocardiography and echocardiography were normal.

Genetic testing failed to demonstrate pathogenic variants in mitochondrial DNA (mtDNA), or the nuclear genes *POLG* and *TWNK.* Subsequent whole genome sequencing and identified homozygous pathogenic variants at the *SPG7* c.2182–2A>G splice site.

## Discussion

We report a case with pathogenic variants in *SPG7* and a family history with pseudodominant inheritance pattern rather than autosomal recessive. This case demonstrates a potential pitfall for parsimonious genetic testing in disorders with high carrier prevalence and the importance of cascade testing to resolve inconsistent inheritance patterns.

Pseudodominant inheritance – where an autosomal recessive condition mimics dominant inheritance either due to a high carrier frequency, unidentified consanguinity or by chance – can confound traditional targeted genetic testing strategies. This phenomena has been documented in other recessive genetic ataxia syndromes such as Friederich’s Ataxia [5] and illustrates the pitfalls of assuming vertical transmission is autosomal dominant. Whilst autosomal dominant *SPG7* disease has been suggested in literature [6, 7], these studies lack whole genome sequencing and may have missed pathogenic deep intronic variants driving compound heterozygosity which are not flagged in targeted sequencing [8]. Whilst true autosomal dominant *SPG7* disease remains possible, without confirmatory testing these reports would also support a pseudodominant hypothesis. Misidentification of asymptomatic carriers has important implications for family and reproductive counselling.

The genetic differential diagnosis of spasticity, ataxia and CPEO includes autosomal dominant nuclear-encoded genes including *ATXN3* (SCA3), *AFG3L2* (SCA28), *TWNK* (PEO1), *POLG*, as well as autosomal recessive genes such as *SPG7*, encephalopathic CoQ10 deficiency and *AFG3L2*-SCAR.

In Case 1, cascade testing confirmed pseudodominant inheritance with compound heterozygous inheritance of maternal *SPG7* c.2182–2A>G and c.1529C>T p.(Ala510Val) from a paternal carrier.

Pseudodominant inheritance of *SPG7* has been reported throughout literature, however its prevalence is unknown. Van Gassen et al., 2012 reported one instance of pseudodominant inheritance across 37 Dutch families with *SPG7* [9], whilst the New Zealand cohort reported by Roxburgh et al. 2012 identified 1 case of pseudodominant inheritance out of 20 families with *SPG7* [4]. De la Casa-Fages et al., described two unrelated families out of 26 in a Spanish cohort demonstrating pseudodominance [10]. All reported cases involved an asymptomatic heterozygous carrier parent with p.(Ala510Val), which is consistent with our family. This pattern is a result of high population frequency of p.(Ala510Val), yet it is infrequently captured in the literature, reinforcing the value of segregation analysis and biallelic confirmation rather than reliance on apparent vertical transmission.

*SPG7* c.1529C>T p.(Ala510Val) has a high background carrier frequency of 3–4% identified in European genomic studies [11, 12], resulting in its potential for pseudodominant inheritance patterns. Whilst population-specific frequencies are not available in local Australian or New Zealand cohorts, given the predominantly British, Irish and broader European ancestry, a comparable carrier burden is expected [13, 14]. It is therefore consistent that p.(Ala510Val) was reported as the most common cause of adult-onset neurogenetic disease in a New Zealand series of patients of British ancestry [4], and *SPG7* was a leading cause of SPAST-negative hereditary spastic paraplegia in an Australian cohort [15]. This finding is consistent with other European HSP cohorts [15–17].

*SPG7* is found on chromosome 16q24.3 and consists of 17 exons to encode a 795 amino-acid long mitochondrial protein, paraplegin [1]. This forms a hexameric complex with AFG3L2 to maintain mitochondrial proteostasis. Case 1 possessed two pathogenic variants predicted to differ markedly in residual function – firstly, the c.2182–2A>G variant localises to the canonical splice acceptor site for exon 17, with in-silico prediction tool spliceAI suggesting high likelihood of acceptor splice site impairment [18]. Skipping of exon 17 results in non-expression of the C-terminus domain and prevents hexameric assembly with AFG3L2, which we predict to result in severe functional impairment. In contrast, the c.1529C>T p.(Ala510Val) variant involves the AAA-ATPase domain, impairing but not abolishing the recognition misfolded mitochondrial proteins [4]. The latter variant drives a phenotype with milder severity, which suggests a gene-dosage functional gradient of paraplegin based on the inherited variants. Within Case 1’s family, we see a natural example - homozygosity for the null-like allele (Case 2) confers the greatest loss of function and the earliest, most severe disease; the compound heterozygous combination of a null-like and a hypomorphic allele (Case 1) yields an intermediate level of residual function. At a cellular level, neurons derived from in-vitro induced pluripotent stem cells from Case 1 (reported as Patient 3 in Wali et al., 2023) did not demonstrate quantitatively reduced paraplegin expression, implying functional impairment of the protein [19].

Across cohorts, compound heterozygotes carrying p.(Ala510Val) tend to show earlier onset by 4.8 years and greater ataxia and cerebellar phenotype severity than p.(Ala510Val) homozygotes [3, 20], positioning Case 1 between biallelic-null (c.2182–2A>G, Case 2) and biallelic-missense (p.Ala510Val) disease and underscoring that the nature of the second allele materially shapes the clinical picture in compound heterozygotic p.(Ala510Val)-related disease. This mechanistic correlate is consistent with Case 1’s earlier symptom onset relative to typical p.(Ala510Val) homozygotes.

*SPG7* disease is a late-manifesting cause of HSP, with age at onset ranging from age 30–40 [21], with the c.1529C>T p.(Ala510Val) pathogenic variant often presenting later with predominant ataxia, as in Case 1. Affected individuals tend to demonstrate a lower limb-predominant cerebellar-spastic ataxia overlap, as well as dysarthria, ocular myopathy [22, 23] and urge incontinence [16, 21]. Rarer presentation such as parkinsonism [24] and motor-neuron disease [25] have also been reported.

## Conclusion

The accurate and timely diagnosis of genetic disorders is further complicated by incomplete penetrance, phenotypic variability and a wide spectrum of disease [4]. As such, the importance of whole genome sequencing for these presentations is increasingly recognised [26, 27]. We also note the importance of cascade testing in resolving ambiguous inheritance patterns and its implications for genetic counselling. In such cases, the focus of reproductive counselling can therefore be shifted from prenatal genetic testing to carrier testing of the unaffected partner, with substantial reduction in cost and risk.

## Data Availability

No datasets were generated or analysed during the current study.
